# Interpreting Tendon Rupture Risk and Trends in Surgical Repair With Testosterone Therapy: Response

**DOI:** 10.1177/23259671261466187

**Published:** 2026-09-30

**Authors:** Al-Hassan Dajani, Timothy P. Liu, Thomas J. Kremen

**Affiliations:** Los Angeles, CA, USA

**Author Response:**

We thank the reader for taking the time to engage with our work and are grateful for their comments. We hope to address the reader’s concerns with this response.

The first concern is regarding the calculations of the reported tendon rupture rates in Table 1. The discrepancy between the reader’s proposed rates (calculated using 7222 testosterone users and 1,177,795 nonusers as denominators for all data points) and the rates we report is due to i2B2 database particularities. Specifically, the numbers shown in the flowchart in Figure 1 and in each row of Table 1 were obtained from separate queries to the i2B2 system, as described in our Methods section. The “denominators” or total testosterone users and nonusers for each row/cohort in Table 1 are individually retrieved for each query. Thus, the rupture rate may slightly vary between queries, due to a combination of exclusion criteria, changes in institutional data availability, interval addition of newly available data, and inbuilt addition/subtraction of a random number of patients in each search to preserve anonymity. However, this is a limitation of any database study that involves the use of an automated query system.

The second concern proposes potential confounding variables that may affect our findings. While we agree with the confounders suggested by the reader, we emphasize that these factors are already acknowledged and discussed in the Discussion section of our work. For example, the reader mentions that this study “could not account for vigorous exercise, resistance training, or nonprescription anabolic steroid use.” We already address in the discussion, stating that “we cannot rule out confounding variables such as vigorous exercise or weightlifting, as well as the presence of patients with chronic tears presenting with acute symptoms in the TRT (testosterone) cohort.” Similarly, we also discuss that our data were limited due to the inability to obtain “granular pharmacologic details” to understand the details of “patients with unknown dosages or frequency.” These are limitations that can sometimes be inevitable when conducting retrospective institutional research, especially with emerging therapies such as testosterone replacement.

The third concern is regarding the heterogeneity of testosterone replacement therapy dosing in our patient cohorts. We currently acknowledge this shortcoming in the Discussion section. We also attempted to mitigate this issue before publication by compiling a table of all the routes, frequencies, and doses of testosterone used in this study cohort to provide some dosing context. We emphasize in the paper that not all of these administration routes and doses are directly comparable, which prevents direct normalization and comparison of testosterone treatment between patients. We do not have blood testosterone levels—these data can be difficult to retrieve retrospectively, as not all patients have routine testosterone level monitoring. While we agree that blood testosterone levels and controlled dosing would be useful data to obtain in future studies, we wish to reiterate that these granularities are not easily explorable with this study design.

The final concern is the lack of clarity on why testosterone replacement therapy users underwent fewer rotator cuff repairs. We agree with this point and already state in the manuscript that we do not have a clear reason for this finding. We provide one hypothesis in the Discussion section that it may be due to testosterone users being offered nonsurgical treatment at higher rates due to their complication profile. We also reference that “previous studies reported a >20-fold increased risk of requiring revision RCR.” We appreciate that the reader provides several examples of factors that surgeons could consider for tailoring treatment; however, we did not analyze surgeon preferences at our institution as part of this retrospective study. We highlight the need for future research to help clarify these trends in the Discussion section.

In summary, we thank the reader for raising these concerns. While many of the limitations are unfortunately inherent to the retrospective design of this study, they are still important clinical questions that would benefit from future research. We also emphasize that we have preemptively addressed many of these limitations in the manuscript within the Discussion section. We welcome any further inquiries and are grateful for the chance to discuss our work.


Al-Hassan Dajani, BS, Timothy P. Liu, MD, and Thomas J. Kremen, Jr., MD *Los Angeles, CA, USA*



